# Pediatric lumbar disc herniation: a report of two cases and review of the literature

**DOI:** 10.1186/s40001-022-00696-x

**Published:** 2022-06-03

**Authors:** Yi Wang, Yan Xu, Guogang Tian, Guogang Dai

**Affiliations:** 1 Cervicodynia/Omalgia/Lumbago/Sciatica Department 2, Sichuan Province Orthopedic Hospital, 132 West First Section First Ring Road, Chengdu, 610041 Sichuan China; 2 Experiment Teaching Center for Preclinical Medicine, Chengdu Medical College, Xindu District, 783 Xindu Avenue, Chengdu, Sichuan China

**Keywords:** Pediatric, Lumbar disc herniation, Nonsurgical treatment

## Abstract

**Background:**

Lumbar disc herniation (LDH) is not a common condition in children. Most reports on pediatric LDH concern the outcomes of surgeries performed in children in whom nonsurgical treatment failed while the outcome of nonsurgical treatment of LDH in children was rarely reported.

**Cases presentation:**

Case 1: a 10-year-old girl presented with back pain and sciatica in her left leg for over 3 months. The physical examination revealed exacerbation of back pain by waist extension or flexion, and a positive Lasegue’s sign was revealed in her left leg. Magnetic resonance imaging (MRI) revealed lumbar disc herniation at the L5/S1 level. She was diagnosed with LDH. After receiving nonsurgical treatment of traditional Chinese medicine (TCM) for 30 days, the girl had mild low back pain and sciatica and the symptoms had resolved completely at the 3-month follow-up. There was no recurrence within the following 2 years. MRI performed 30 months later revealed that the herniated disc did not shrink significantly. However, she was totally asymptomatic at the follow-up performed 30 months later. Case 2: a 13-year-old boy presented with sciatica in his left leg for over 3 months. The physical examination revealed that Lasegue’s sign was positive in the left leg, the level of muscle strength in the left ankle plantar flexors was grade 4. MRI revealed a lumbar disc herniation at the L5/S1 level. He was diagnosed with LDH. The boy underwent 2 weeks of TCM treatment, and exhibited a favorable outcome: only mild pain was noticed in his left buttocks after walking for more than 15 min. He was asymptomatic at the 3-month follow-up and there was no recurrence within the next 3 years. MRI scan performed at 40 months later showed no significant resorption of the herniated disc. However, he was totally asymptomatic at the follow-up performed 40 months later.

**Conclusions:**

For the nonsurgical treatment of pediatric LDH, resorption of herniated discs is not necessary for favorable long-term outcomes, and children with symptomatic LDH may become asymptomatic without resorption.

## Background

Lumbar disc herniation (LDH) is not a common condition in children. The overall incidence of LDH in children is unknown, and the diagnosis of LDH is usually delayed in children compared with adults [1, 2]. Since the first case of pediatric LDH was reported in 1945 [3], many surgeons have reported pediatric patients undergoing surgery for LDH [4–11], and pediatric patients have been reported to constitute 0.4–15.4% of surgically treated patients with LDH [2]. Most reports on pediatric LDH concern the outcomes of surgeries performed in children in whom nonsurgical treatment failed while the outcome of nonsurgical treatment of LDH in children was rarely reported [4–11]. We reported a follow-up study of two pediatric LDH cases treated with nonsurgical treatment of traditional Chinese medicine (TCM) to present the long-term outcomes of LDH in children.

## Cases presentation

### Case 1

**Fig. 1:**
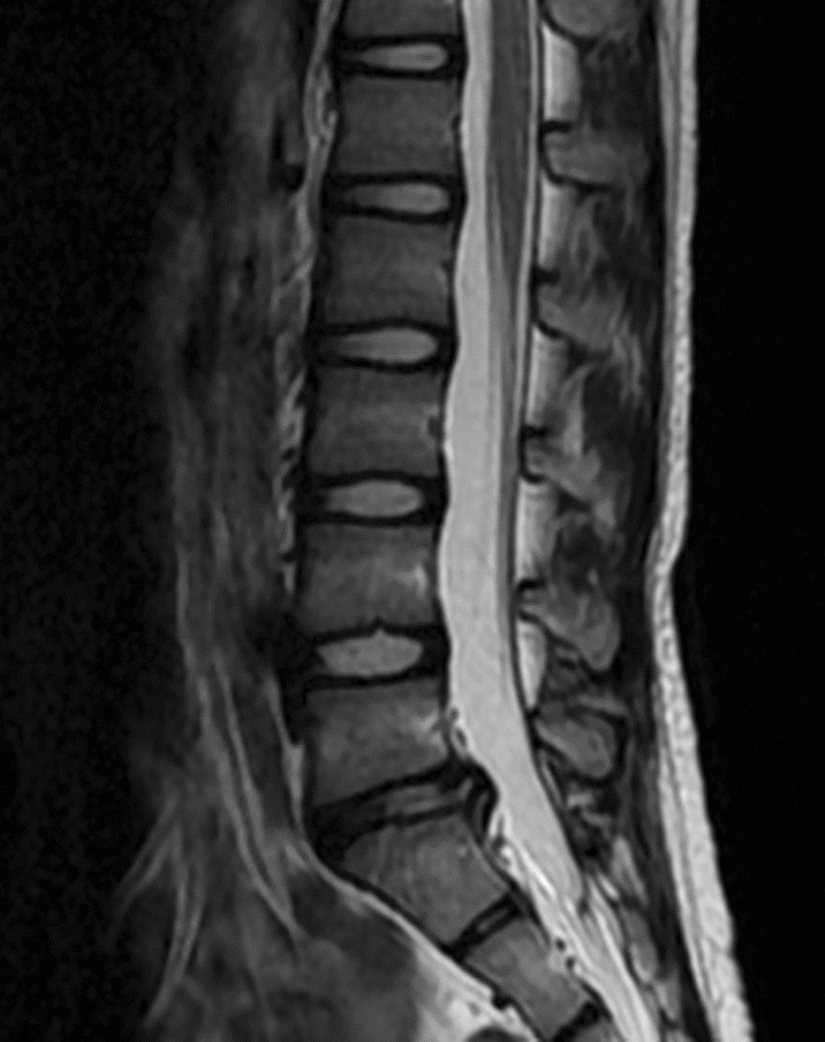
MRI of the lumbar spine obtained at the 10-year-old girl’s initial visit revealed Schmorl’s node at L4 and S1, platyspondyly of the lumbar vertebrae, and a lumbar disk herniation at the L5/S1 level

**Fig. 2:**
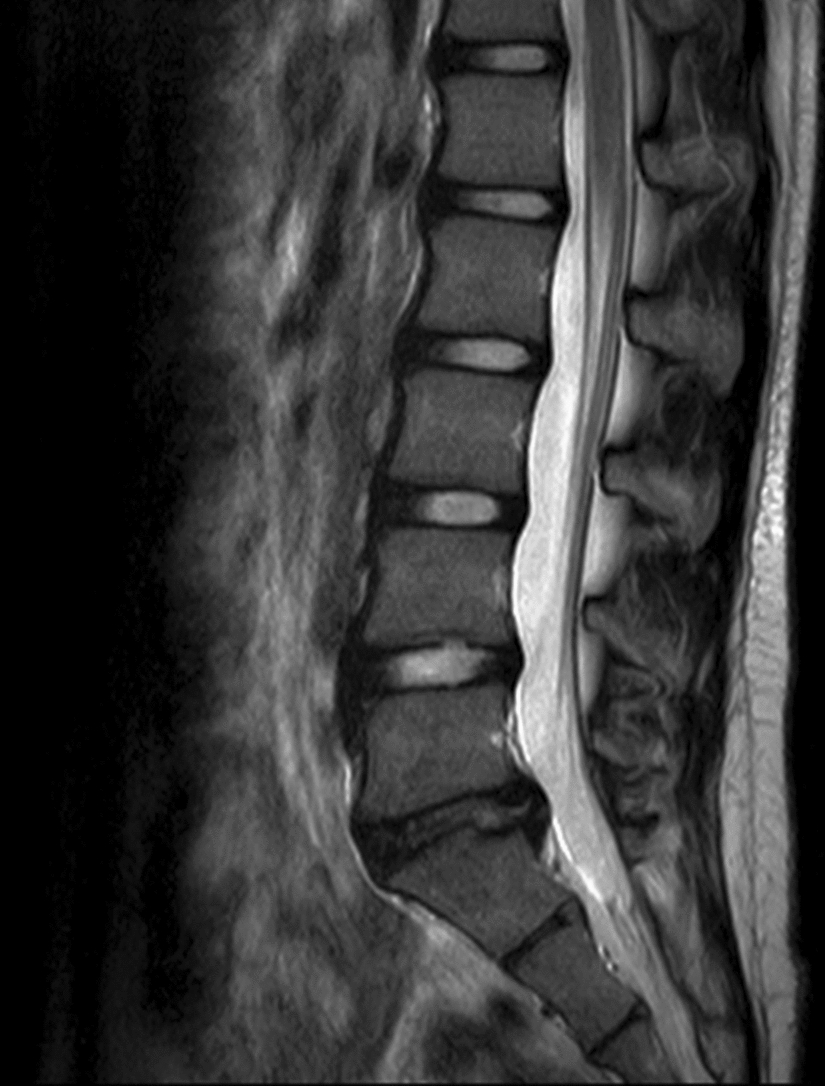
MRI obtained at the girl’s 30-month follow-up showed no significant changes in Schmorl’s node at L4 and S1, platyspondyly of the lumbar vertebrae, and no significant resorption of the herniated L5/S1 lumbar disc

A 10-year-old girl presented with back pain and sciatica in her left leg for over 3 months. The girl did not experience trauma, and she began to feel pain when she was sitting on a bench doing homework. She was hospitalized for persistent pain and limp caused by pain when walking. The physical examination revealed exacerbation of back pain by waist extension or flexion, and a positive Lasegue’s sign was revealed in her left leg. There was no sign of hypoesthesia or decreases in muscle strength or bowel or bladder dysfunction. Magnetic resonance imaging (MRI) revealed Schmorl’s node at L4 and S1, platyspondyly of the lumbar vertebrae, and a lumbar disc herniation at the L5/S1 level (Fig. 1). The girl was diagnosed with LDH at the L5/S1 level. Nonsurgical treatment of TCM was administered for 30 days, which included bed rest, electroacupuncture, Chinese Tuina, Chinese herbal fumigation, and moxibustion at the lumbosacral area. She had mild low back pain and sciatica when she was discharged from the hospital, and the symptoms had resolved completely at the 3-month follow-up. There was no recurrence within the following 2 years. MRI performed 30 months later revealed that the Schmorl’s node and the platyspondyly of the lumbar vertebrae did not change significantly, and that the herniated disc did not shrink significantly (Fig. 2). However, she was totally asymptomatic at the follow-up performed 30 months later.

### Case 2

**Fig. 3:**
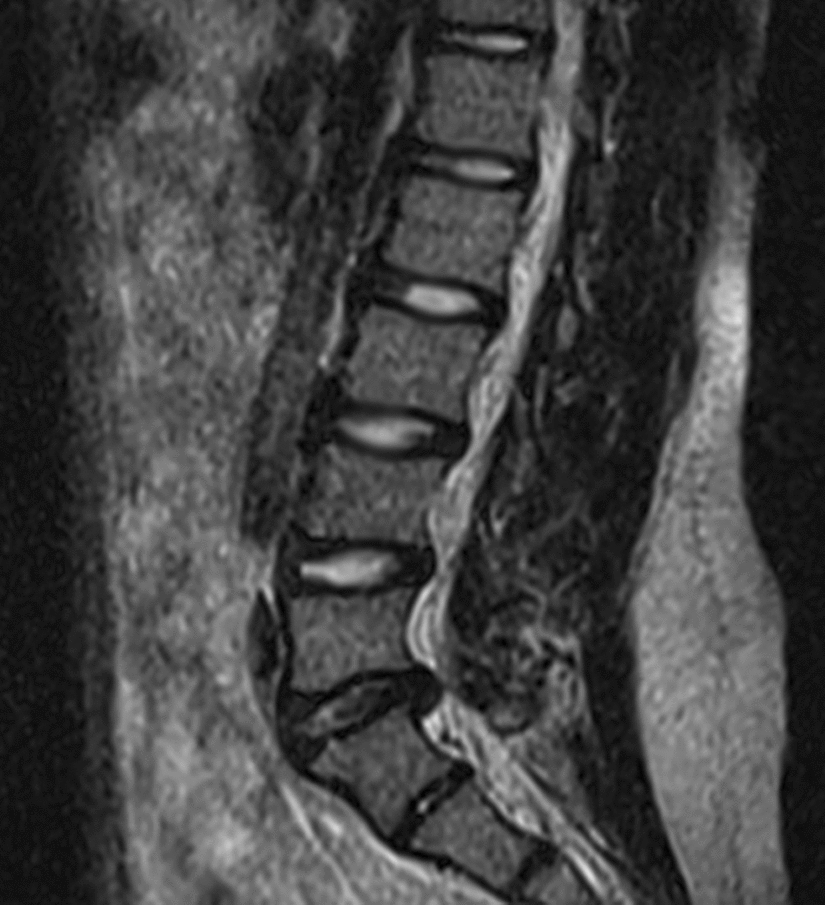
MRI of the lumbar spine obtained at the 13-year-old boy’s initial visit revealed platyspondyly of the lumbar vertebrae, and a lumbar disk herniation at the L5/S1 level

**Fig. 4:**
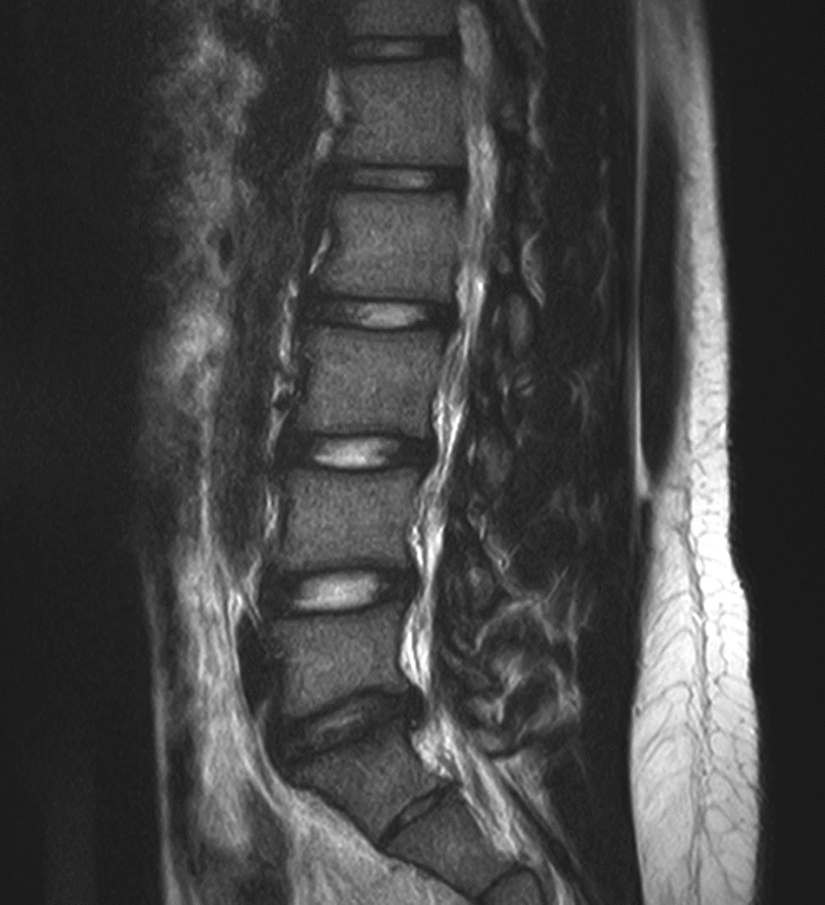
MRI obtained at the boy’s 40-month follow-up showed no significant changes of platyspondyly of the lumbar vertebrae and no significant resorption of the herniated L5/S1 lumbar disc

A 13-year-old boy was hospitalized for sciatica in his left leg for over 3 months. He felt pain in the distribution of the sciatic nerve when sitting, standing, and walking. The physical examination revealed that Lasegue’s sign was positive in the left leg, the level of muscle strength in the left ankle plantar flexors was grade 4, and the levels of lower limb sensation and bowel and bladder function were normal. MRI revealed platyspondyly of the lumbar vertebrae and a lumbar disc herniation at the L5/S1 level (Fig. 3). The boy was diagnosed with LDH at the L5/S1 level. The patient underwent 2 weeks of nonsurgical treatment of TCM, as case 1 did, and exhibited a favorable outcome: only mild pain was noticed in his left buttocks after walking for more than 15 min. He was asymptomatic at the 3-month follow-up and did not experience back pain or sciatica within the next 3 years; the MRI scan performed at 40 months after the first MRI scan showed no significant changes of platyspondyly of the lumbar vertebrae and no significant resorption of the herniated disc (Fig. 4).

## Discussion and conclusions

LDH is based on disc degeneration, which occurs with ageing [12]. Early histopathologic degeneration of the lumbar disc initiates at the age of 11 [13], and the water content of the disc begins to decrease in early childhood [14]. Other risk factors for LDH include trauma, physical loading, obesity, socioeconomic status, and genetic and behavioral influences [14–17]. The main causes of LDH in children are traumatic and sports injuries [1]. The youngest child reported to have LDH was 13 months old [18], and there are reports on cauda equina syndrome symptoms caused by LDH in children [19, 20].

The prevalence of Schmorl’s node in children and adolescents is 2.68%, whether symptomatic or asymptomatic [21]. In all age groups of patients with suspicion of lumbar lesions, the incidence of Schmorl’s node is the highest in teenagers, at 57%, and most of these teenagers complain about back pain [22]. In the present study, the child presented with Schmorl’s node and platyspondyly of the lumbar vertebrae, which is mostly related to skeletal dysplasia. In skeletal dysplasia, the vertebral body is prone to intraosseous fractures, resulting in Schmorl’s node formation and causing pain. As the intraosseous fractures heal, Schmorl’s nodes become asymptomatic [23]. Although Schmorl’s node was present in case 1, there were no inflammatory changes in the vertebral body marrow on MRI, and we believe it is not a cause of pain, analogous to old fractures.

In skeletal dysplasia, rupture of the annulus fibrosus is also more likely to present in children, resulting in disc herniation and leading to back pain or sciatica. Pediatric LDH is managed similarly to adult LDH, which includes nonsurgical and surgical treatment [1]. For adult patients with LDH, surgery relieves pain quickly within a short period, with an incidence of complications of up to 12.5% [24], while nonsurgical treatment is usually performed for more than 6 months [25, 26]. Randomized clinical trials have suggested that surgery relieves pain faster than nonsurgical treatment at 6 months [25–27]; subsequently, the results of the two treatments tend to be similar between 6 and 12 months after the start of treatment [25, 27]. A one year after treatment, resorption of LDH was observed in 91% of nonsurgically treated patients, while LDH was unchanged or enlarged in 5% of patients who underwent surgery; not all patients with resorption achieved favorable outcomes, while not all patients with unchanged or enlarged LDH had unfavorable outcomes [28]. From 1–2 years, there was no difference between the outcomes of the patients treated by the two methods [25, 27, 28]. However, a limitation of these trials was nonadherence to the treatment assignment, as patients sometimes received both treatments or switched groups, and the conclusions of these trials need to be interpreted with caution. One randomized clinical trial suggested that both surgically and nonsurgically treated patients achieved substantial relief within 2 years, with no statistically significant difference between the patient groups [29]. In a cohort study, surgery was reported to improve LDH better than nonsurgical treatment within 2–4 years according to combined as-treated analysis [30, 31]. There was no consensus on whether surgical treatment or nonsurgical treatment for LDH is more efficacious in adults, and there is no evidence that these conclusions apply to children.

Surgery is not a first-line treatment for children with LDH; it is performed only when progressive neurological deficits and bowel or bladder dysfunction are present or nonsurgical treatment has failed [32]. Nonsurgical treatment failed in most pediatric patients reported in the literature, and thus, surgery was performed for these children [4–11]. In the long term, one mechanism of relief after nonsurgical treatment is resorption of the herniated disc, which reduces the pressure on the nerve root, the source of inflammation and the immune response. Resorption is a common phenomenon in the natural history of LDH [33, 34], and most herniated discs shrink over time [35]. Our previous study revealed that the overall incidence of resorption of symptomatic LDH after nonsurgical treatment was 63% [36]. However, pediatric LDH is less likely to resorb than is adult LDH [37], which may explain why children were less responsive to nonsurgical treatment than adults.

Few studies have reported nonsurgical treatment for children with LDH to be successful. The commonly used nonsurgical treatments for pediatric LDH are similar to those for adults, including bed rest, rest, physical therapy, NSAIDs, and analgesic agents [32]. Many studies have confirmed that TCM is effective in treating LDH in adults [38–41]. A randomized controlled clinical trial suggested that TCM has good clinical efficacy for LDH by improving local inflammation, circulation, and edema of the nerve root [41]. Yu et al. suggested that nonsurgical treatment of TCM was effective for ruptured LDH and can promote herniated disc resorption [40]. Acupuncture and Chinese Tuina relieve pain and improve lumbar dysfunction in LDH [38, 42]. Animal experiments have shown that electroacupuncture reduces the release of inflammatory cytokines, including TNF-α IL-1β, IL-10 and TGF-β [39]. Our previous study suggested that integrated TCM relieved pain due to LDH by downregulating inflammation-related factors, such as TLR5, IL1RN and IL-27 [43]. However, the underlying mechanism by which TCM decreases the symptoms of LDH remains largely unknown. TCM has seldom been reported for the treatment of pediatric LDH. In the present study, two children with LDH achieved favorable outcomes after nonsurgical treatment of TCM treatment; there was no recurrence or aggravation within a period of more than 2 years, and both patients were asymptomatic at the last follow-up, although no resorption of the herniated discs was observed on MRI (in the present report “significant resorption” refers to a reduction in size of more than 50% as defined by Takada [44]).

In conclusion, for the nonsurgical treatment of pediatric LDH, resorption of herniated discs is not necessary for favorable long-term outcomes, and children with symptomatic LDH may become asymptomatic without resorption after nonsurgical treatment.

## Data Availability

Not applicable.

## Abbreviations

LDH: Lumbar disc herniation
TCM: Traditional Chinese medicine
MRI: Magnetic resonance imaging
